# Restoring Mitochondrial Function While Avoiding Redox Stress: The Key to Preventing Ischemia/Reperfusion Injury in Machine Perfused Liver Grafts?

**DOI:** 10.3390/ijms21093132

**Published:** 2020-04-29

**Authors:** Julia Hofmann, Giorgi Otarashvili, Andras Meszaros, Susanne Ebner, Annemarie Weissenbacher, Benno Cardini, Rupert Oberhuber, Thomas Resch, Dietmar Öfner, Stefan Schneeberger, Jakob Troppmair, Theresa Hautz

**Affiliations:** Department of Visceral, Transplant and Thoracic Surgery (VTT), Daniel Swarovski Research Laboratory (DSL), Medical University of Innsbruck (MUI), Innrain 66, A-6020 Innsbruck, Austria

**Keywords:** ischemia/reperfusion injury, liver transplantation, redox stress, mitochondrial dysfunction, reactive oxygen species, machine perfusion

## Abstract

Mitochondria sense changes resulting from the ischemia and subsequent reperfusion of an organ and mitochondrial reactive oxygen species (ROS) production initiates a series of events, which over time result in the development of full-fledged ischemia-reperfusion injury (IRI), severely affecting graft function and survival after transplantation. ROS activate the innate immune system, regulate cell death, impair mitochondrial and cellular performance and hence organ function. Arresting the development of IRI before the onset of ROS production is currently not feasible and clinicians are faced with limiting the consequences. Ex vivo machine perfusion has opened the possibility to ameliorate or antagonize the development of IRI and may be particularly beneficial for extended criteria donor organs. The molecular events occurring during machine perfusion remain incompletely understood. Accumulation of succinate and depletion of adenosine triphosphate (ATP) have been considered key mechanisms in the initiation; however, a plethora of molecular events contribute to the final tissue damage. Here we discuss how understanding mitochondrial dysfunction linked to IRI may help to develop novel strategies for the prevention of ROS-initiated damage in the evolving era of machine perfusion.

## 1. Introduction

Liver transplantation (LT) is the most efficient therapy for end-stage liver disease. The early outcome after LT has improved over the past years. In experienced centers, an 85–95% 1-year patient survival rate can be achieved [1]. However, wider application of LT is limited by significant organ shortage. In the light of this, organs from extended criteria donors (ECD) are increasingly considered for transplantation. The preexisting damage puts these organs in a state where they seem particularly susceptible to the impact of ischemia and reperfusion.

Restriction of blood supply (ischemia) followed by restoration of perfusion and reoxygenation (reperfusion) initiates a cascade of events in the transplanted organs, which ultimately leads to severely impaired organ function. While static cold storage (SCS) remains the standard for liver preservation, massive accumulation of metabolites derived from anaerobic respiration during the ischemic phase poses a hazard upon reperfusion. There is accumulating evidence, that gentle rather than abrupt rewarming of organs may ameliorate the events by significantly reducing succinate-driven reactive oxygen species (ROS) production and increasing adenosine triphosphate (ATP) levels. Ex vivo machine perfusion (MP) of livers may help preventing mitochondrial and tissue damage. Understanding the alterations leading to organ damage at the molecular levels and the possibility to optimize perfusion protocols to significantly minimize true ischemia times hold significant potential to limit organ damage in transplantation and beyond [2,3].

## 2. Mechanisms of Ischemia/Reperfusion Injury (IRI)

**Figure 1:**
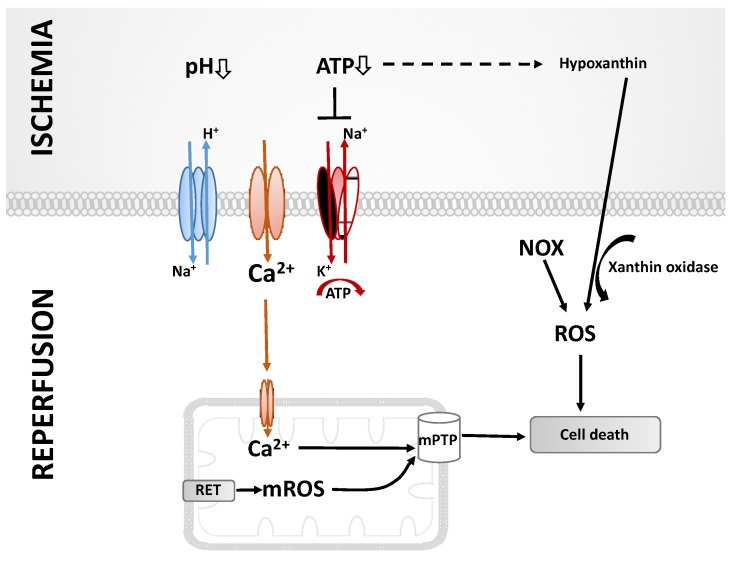
Molecular events leading to ischemia and reperfusion injury (for details see text). ATP: adenosine triphosphate, ROS: reactive oxygen species, mROS: mitochondrial reactive oxygen species, NOX: NADPH oxidases, RET: reverse electron transport, mPTP: mitochondrial permeability transition pore.

The interruption of blood and oxygen supply during organ transplantation remains a part of the routine. To minimize the negative impact on the outcome, ischemia times are kept at a minimum and organs are stored on ice. Because of restricted oxygen supply, the anaerobic metabolism leads to decreased intracellular ATP levels. Anaerobic glycolysis results in the production of lactic acid, thereby resulting in acidosis (Figure 1). Elevated H^+^ levels stimulate the Na^+^/H^+^ exchange and, in turn, intracellularly increased Na^+^ concentrations. Moreover, the ATP-dependent Na^+^/K^+^ and Ca^2+^ pumps fail during ischemia, resulting in an intracellular accumulation of Ca^2+^ [4,5,6]. Blood reperfusion does not immediately restore normal conditions, but rather aggravates the situation. A major event during this vulnerable phase is the production of excessive amounts of ROS, including superoxide anions, hydrogen peroxide and hydroxyl radicals, resulting in aberrant cell signaling, damage to biomolecules, inflammation and cell death, culminating in a decline of organ function [7]. The mitochondrial electron transport system (ETS) is a major source of ROS under these conditions; however, other mitochondrial and non-mitochondrial sources of ROS also contribute to IRI [7,8]. Damage to biomolecules and ROS-mediated signaling also activate innate immune responses, and eventually lead to fibrosis and the deterioration of organ function.

## 3. Sources of ROS

**Figure 2:**
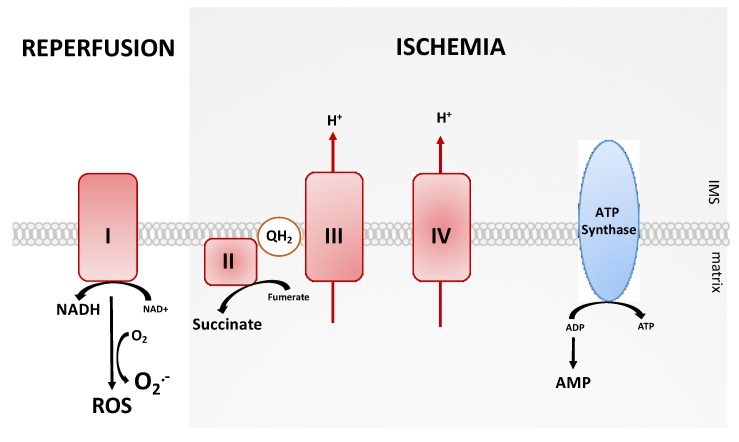
The mitochondrial reverse electron transport (RET) as the main source of reactive oxygen species (ROS) production during ischemia and reperfusion. NAD/NADH: nicotinamide adenine dinucleotide, ADP: adenosine diphosphate, ATP: adenosine triphosphate, IMS: intermembrane space.

Mitochondrial ROS (mROS) are generated during early reperfusion [9] and complex I of the mitochondrial ETS seems to be the most important source. Pharmacological inhibition, e.g., by rotenone [10] or amobarbitol [11], significantly decreases the generation of superoxide. mROS production by complex I can occur through conventional forward electron transport. In the course of IRI, however, the reverse electron transport (RET) mainly contributes to mROS production (Figure 2). The ischemic phase already primes the tissue for the subsequent damage [7]. Reducing equivalents are generated to provide electrons for RET, which are in turn required for mROS production upon subsequent reperfusion [9,12,13]. The predominant mitochondrial metabolite accumulating during ischemia is driven by the reverse operation of succinate dehydrogenase (SDH). Pharmacological inhibition of SDH significantly reduces mROS production [14], which in turn results in the reduction of the Coenzyme Q (CoQ) pool, leading to proton pumping by mitochondrial complexes III and IV. Together with the inhibition of the ATP synthase complex, an increase in proton motive force occurs [7].

Even if complex I may be the main source of mROS production during IRI, there is growing evidence that complexes II and III also contribute to mROS generation. Mitochondria need to be polarized for mROS production by complex I. Opening at the mitochondrial permeability transition pore (mPTP) leads to depolarization and limits complex I mROS production. In a situation where simultaneous inhibition of complex III by antimycin is induced, sustained mROS production by complex II is observed [15]. Inhibition of complex II by atpenin A5 leads to ceased mROS generation by complex I. Persistent mROS production identifies complex III as another possible source of mROS generation [16].

ROS production is not limited to the mitochondria. Xanthine oxidoreductase (XOR) is an enzyme which catalyzes the final two steps of purine catabolism and exists in two isoforms, which are interconvertible: Xanthine oxidase (XO) and xanthine dehydrogenase (XDH). The latter is dominating under physiological conditions, whereas XO contributes to the generation of ROS upon IRI. During the ischemic phase, hypoxanthine accumulates in the ischemic tissue through degradation of ATP. Following reperfusion, XO catalyzes the conversion of hypoxanthine in the presence of tissue oxygen to uric acid, thereby releasing superoxide, which may be dismutated to hydrogen peroxide [17].

Moreover, also nicotinamide adenine dinucleotide phosphate (NADPH)-dependent oxidases are sources of ROS. It has been shown that reduced oxidative phosphorylation results in elevated levels of NADPH [18]. NADPH oxidases are multiprotein complexes, consisting of seven members (named NOX 1-7). These complexes catalyze the electron transport from NADPH to oxygen and thereby result in superoxide anion generation. During hypoxia, tissue-infiltrating neutrophils contribute to ROS production through the NADPH oxidase isoform NOX2 [3].

## 4. Mitochondrial Damage and Dysfunction Following Ischemia and Reperfusion

ROS are not detrimental per se, and physiological levels are actually required for cellular homeostasis. ROS induce reversible post-translational protein modifications in order to regulate signaling pathways [19]. Diverse signaling proteins respond to the production of ROS, including mitogen-activated protein kinases (MAPKs), nuclear factor kappa B (NF-κB), Janus kinase/signal transducers (JAK/STAT), Toll-like receptors (TLR) and nitric oxide (NO) signaling [4,20]. Cytoplasmic signaling regulates, e.g., the activation of the inflammasome, expression of adhesion molecules or various cell death pathways—all of which are necessary for the development of IRI. Moreover, cytochrome *c* release is enabled, which triggers the activation of caspase-9 and thus induces apoptotic cell death via activation of caspase-3 [21,22]. Further to cytochrome *c*, the second mitochondrial-derived activator of caspase/direct IAP inhibitor of apoptosis binding protein with low PI (Smac/DIABLO), high-temperature requirement protein A2 (HtrA2), endonuclease G or apoptosis-inducing factor (AIF) are released and may induce apoptosis [23]. Due to ATP depletion, necrotic cell death is induced. Necrosis is characterized by the rupture of the plasma membrane, as it occurs during reperfusion, which leads to swelling of the cells, the release of damage-associated molecular patterns (DAMPs), and the induction of inflammation. Necroptosis has also been acknowledged for its role in IRI as a regulated form of necrosis [24]. The most critical regulators of necroptosis are receptor-interacting protein kinases 1 and 3 (RIPK1/RIPK3) [25]. Damaged mitochondria can be removed by mitochondria-selective autophagy (mitophagy) in order to keep the remaining hepatocytes viable. Mitophagy may have a protective role in this setting, since mROS production may be limited [3,26]. Beclin-1 is a protein, which stimulates autophagy. Overexpression of Beclin-1 has been shown to reduce IRI-induced cell death [3]. However, the data are conflicting, since other research groups found beneficial effects of inhibiting autophagy [9].

## 5. Liver-Specific Aspects of IRI

With regard to IRI, especially liver sinusoidal endothelial cells (LSECs), are highly vulnerable during the cold ischemic phase. These cells play a major role in vascular homeostasis and immune function. Upon reperfusion LSECs express a multitude of cytokines as well as DAMPs, while an imbalance of NO and endothelin (ET) results in luminal narrowing, thereby negatively impacting on the microcirculation [27,28]. The DAMPs released can be recognized by TLR expressed on Kupffer cells (KCs), leading to activation of these resident macrophages in the liver. Additionally, KCs may be stimulated by the complement mediators C3a and C5a [3]. Activated KCs secrete a battery of cytokines. Tumor necrosis factor α (TNFα), promotes the expression of intracellular adhesion molecule 1 (ICAM1) and vascular adhesion molecule 1 (VCAM1). This allows platelets to adhere to LSECs, hence initiating apoptosis of these cells [29,30]. However, TNFα can also induce apoptosis by binding to TNF receptor (TNFR) on hepatocytes and subsequent activation of NF-κB [31]. Moreover, interleukin 1β (IL-1β) and TNFα promote the infiltration of neutrophils and subsequently induce ROS production by neutrophils [32]. TNFα also stimulates hepatic stellate cells to build scar tissue, which impacts on organ architecture and subsequently leads to altered organ function [3]. In addition to pro-inflammatory factors, KCs may also secrete the anti-inflammatory cytokine IL-10. IL-10 suppresses the NF-κB pathway and reduces inflammation-related injury by inhibiting the expression of pro-inflammatory factors [31].

## 6. Machine Perfusion—An Innovative Technology for Organ Preservation

In recent decades, the gold standard for the preservation of solid organs for transplantation was SCS at 4 °C [33]. Since organs from ECD are more vulnerable to SCS, MP offers an alternative. However, the beneficial effects are not limited to marginal organs [34,35]. The first successfully transplanted liver after MP by Starzl et al. even dates back to the 1960s [36]. Nevertheless, it took more than four decades for this concept of organ preservation to be implemented in clinical practice, mainly due to logistical issues. During MP, organs are perfused continuously ex vivo, either with supplemented blood or modified colloid solutions, which support microcirculation and the washout of metabolic waste [2,33,37]. A major advantage of MP is the ability to assess the quality and function of organs prior to transplantation, which helps transplant surgeons in the decision-making process of whether to use an organ for transplantation or not. Moreover, the addition of substrates and nutrients could support regeneration during the preservation period. In the future, targeted therapeutic interventions may even allow the recovery of originally declined, marginal donor organs and, thus, help to overcome organ shortage. Different strategies have been applied in MP and classified according to the temperature and oxygen supplementation (Table 1).

### 6.1. Hypothermic Machine Perfusion (HMP) at 4 °C

HMP is performed via the portal vein only [38]. Due to the low temperature, metabolism and energy demand are reduced, hence enabling ATP stores to be conserved [33]. There is no need for an extra oxygen carrier in the perfusion solution, because of oxygen being physically dissolved at 4 °C [39]. However, a protective effect—achieved by utilizing a perfusion solution containing an oxygen carrier, which is known as hypothermic oxygenated machine perfusion (HOPE)—has been reported [40]. A further extension of HOPE is so-called dual hypothermic oxygenated perfusion (D-HOPE)—enabling perfusion via the portal vein and the hepatic artery, which improves blood supply to the bile duct [41,42].

### 6.2. Subnormothermic Machine Perfusion (SNMP) at 20–25 °C

SNMP displays a compromise of HMP and normothermic machine perfusion (NMP), and thus combines the respective advantages of HMP and NMP. The temperature is low enough to achieve sufficient oxygenation without the implicit addition of an oxygen carrier, which enables the restoration of ATP levels. At the same time, the temperature is high enough to allow for the partial viability assessment of the organ [13,43,44].

### 6.3. Normothermic Machine Perfusion (NMP) at 37 °C

During NMP, organs are perfused at 37 °C to simulate near-to physiologic conditions [2]. Thus, the perfusion solution must contain an oxygen carrier and nutrients. Since metabolic functions are fully maintained, NMP is the type of MP most suitable for organ quality assessment prior to transplantation, which is of particular interest in the context of ECD organs. Moreover, the preservation period can be extended and thus offers a therapeutic window for specific interventions to repair marginal organs [33,35,37,45].

### 6.4. Controlled Oxygenated Rewarming (COR)

The most recently described perfusion strategy is COR. By gradually increasing the temperature after a cold perfusion period, it aims to minimize injury, which is normally triggered by a sudden temperature shift. After an initial period of HMP with a subsequent phase of COR to reach NMP conditions, the beneficial effects of HMP and NMP can be combined [46,47].

## 7. Effect of Normothermic Machine Liver Perfusion on IRI

Implementing NMP in the clinical routine of liver transplantation has helped to reduce cold ischemic time in the course of organ preservation; however, cold ischemia cannot be totally averted in most cases. Most often, NMP of donor livers is started after a limited period of conventional SCS, e.g., after organ transportation. In NMP, livers are rewarmed and reperfused on the machine after a period of cold ischemia. While this is an equivalent of in situ reperfusion upon transplantation, the perfusion fluid used for NMP normally does not contain leukocytes and platelets, which are detrimental in the development of IRI [2]. Together with the limited inflammatory response, this may be indicative of a more benign and less harmful reperfusion in this setting.

Several studies in liver grafts have already shown a benefit of NMP on IRI, even though complete prevention of IRI cannot be expected. Zhang et al. [48] conducted a study with reduced-size porcine livers and applied NMP prior to transplantation. Compared to the control group (SCS), serum levels of the proinflammatory cytokines TNFα and IL-6 were significantly decreased in the NMP group post transplantation. These findings are consistent with Schlegel et al. [49] and Jassem et al. [50]. The latter investigated the expression of various inflammation- and tissue regeneration-related genes. The expression was significantly reduced in the human livers preserved with NMP compared to the SCS group. Moreover, NMP may reduce the expression levels of cytochrome *c* and caspase-3, which was observed in the study of Zhang in porcine livers [48]. This suggests a decrease in apoptotic cell death as induced by IRI. A reduction in cell death as by histopathologic analysis of liver biopsies collected after NMP was also confirmed in the rat liver model with subsequent transplantation [49], and in human livers with subsequent transplantation [50].

## 8. Monitoring an Organ During MP: Potential Biomarkers for Quality Assessment

One of the major advantages of NMP compared to SCS is the possibility to monitor an organ prior to transplantation and to assess its quality under close-to-physiological conditions. Therefore, the establishment of reliable markers and parameters suitable for organ viability testing during NMP, hence predicting organ function and transplant outcome, are urgently needed.

### 8.1. Liver Function Parameters

For the liver, bile production may serve as a marker for the viability assessment as reported by Sutton et al. [51], since intact sinusoidal cells, hepatocytes and cholangiocytes are required for bile production. Similar findings have been reported by op den Dries et al. [52]. However, the organs were not transplanted after NMP in these studies. In contrast, Nasralla et al. found no correlation between bile production during NMP and post-transplant liver function in their large clinical study including 220 liver transplantations, where SCS was compared to NMP. Nevertheless, significantly lower levels of aspartate aminotransferase (AST) were found in the NMP group after transplantation [53]. Together with alanine aminotransferase (ALT), AST is considered a biomarker indicative for hepatocyte damage during MP and upon transplantation [51,54]. Moreover, various studies showed that pH, glucose levels and lactate clearance may serve as suitable functional markers for the liver during ex vivo NMP [51,54,55].

### 8.2. Potential Biomarkers for IRI Assessment

In search of biomarkers that are indicative of damage associated with IRI, De Vries et al. [56] recently studied the different cell types released into the perfusion solution during MP in rat livers. Even though they subjected the organs to SNMP, their findings are interesting in the context of possible novel biomarkers for NMP. They found significant differences in the release of LSEC, KCs and stellate cells, which correlated with the severity of the ischemic injury. Karangwa et al. [57] suggested the activation of fibrinolysis during NMP as a suitable parameter for liver IRI. In their study with 12 discarded human livers, they found higher D-dimer (a marker of fibrinolysis) levels for organs with poor function compared to good function. They confirmed these findings by the observation that D-dimer levels correlated with ALT levels. Another potential biomarker for the assessment of IRI may be cell-free microRNAs (miRNA). The release of hepatocyte-derived miRNAs (HDmiRNA) during NMP was shown to correlate with AST levels [58]. However, it must be taken into account that study protocols and donor criteria vary markedly between all the studies mentioned, and further clinical studies with subsequent transplantations and follow-up are urgently warranted to verify these findings.

### 8.3. Mitochondria-Specific Assessment During MP

As protection of mitochondria is crucial to prevent IRI, the assessment of mitochondrial function may help to predict the extent of IRI damage and hence organ performance. Since ATP is depleted during ischemia, measurement of ATP tissue levels during MP is suggested to serve as a reliable mitochondrial function marker. During the preservation phase, the respective MP strategies contribute to the resynthesis of ATP, whereby the recovery of the energy status correlates with the functionality of the organ [13].

Quantification of the DAMPs released also offers a potential parameter for the assessment of mitochondrial function during MP, since the release of DAMPs is induced by IRI. A key member of this group is the high mobility group box-1 protein (HMGB-1) [31]. First experiments in a rat liver study showed decreased levels of HMGB-1 after NMP compared to SCS [49].

A well-established method for assessing mitochondrial function is high-resolution respirometry (HRR), which has already been used to analyse rat [59], pig [60] and human liver biopsy samples [61,62,63]. With adequate HRR protocols, the state of the mitochondrial OXPHOS machinery can be analysed, which reflects the damage to mitochondria during IRI [64]. After titration of specific substrates and inhibitors of the convergent electron pathways leading to the ETS, the oxygen consumption is measured and allows for a distinct evaluation of the respective pathways. The outer mitochondrial membrane integrity can be tested by titration of cytochrome *c*, whereby an increase in oxygen consumption as a response is linked to membrane damage. The coupling status of the oxidation to the phosphorylation reports about inner membrane integrity [61,65,66]. Longitudinal assessment of liver biopsy samples collected during MP and analysed with HRR may provide novel insights into how MP impacts on mitochondrial function of a liver.

## 9. How the Type of MP Impacts on Mitochondrial Function

Mitochondrial activity and, in general, metabolic activity are known to be temperature-dependent. Not surprisingly, different MP types operating at different temperatures, as displayed above, may exert various effects on mitochondrial activity and cellular responses. Karimian et al. [43] compared SNMP and NMP in steatotic human livers and found increased ATP stores during perfusion; however, significantly higher levels were seen in the SNMP group. These findings confirm the per se expected temperature-dependent increase in mitochondrial activity, leading to an elevated ATP turnover in NMP livers and thus to reduced ATP perfusate (serum) levels. However, the higher metabolic activity may be responsible for an enhanced ROS production at physiological temperatures (NMP) [2]. Hence, the idea of combining perfusion techniques operating at different temperatures was triggered by the finding that succinate metabolism already starts with forward electron transport during an initial phase of HOPE. Hence, the major “burst” of ROS production and the subsequent oxidative stress upon reperfusion can be ameliorated [67]. Martins et al. [68] perfused rat livers under mild hypothermic and normothermic conditions and analysed isolated mitochondria. The authors observed reduced oxygen consumption in the presence of succinate and reduced ATP levels in the normothermic group. These findings correlated with their histopathological observations in liver biopsies: In the mild hypothermic group, only intact hepatocytes were found, whereas in the normothermic group a moderate disassociation of the hepatocytes, indicative for cell damage, was revealed. The results of this study may also indicate a superiority of preservation at lower temperatures to prevent damage to mitochondria.

While hypothermic preservation (HOPE) decreases mitochondrial respiration and enhances the ATP pool during preservation, NMP is the preferential preservation technique for viability testing as it restores metabolism under physiologic temperatures and hence allows functional monitoring under close-to-physiologic conditions. The direct effect of NMP and HOPE on liver cell injury have already been tested in rat liver grafts. NMP resulted in a reduced cell infiltration and IRI. This effect was further enhanced by HOPE [49]. In the light of these findings, Boteon et al. [67] suggest combining the advantageous effects of HOPE on cell metabolism with the benefits of NMP in terms of quality and functional organ assessment. In their study including 10 discarded human ECD livers, they combined both techniques. Mitochondrial respiration was downregulated during HOPE, and the tissue ATP levels were increased. During the following NMP, reperfusion injury was significantly reduced. This observation suggests that a combined perfusion protocol attenuates oxidative stress as well as tissue inflammation in liver grafts. In addition, in the follow-up study of this group, where the authors additionally used a haemoglobin-based oxygen carrier (HBOC)-based perfusate to enable an uninterrupted cold-to-warm perfusion of the livers, similar results were observed [69]. Another study where the combined perfusion techniques (HOPE-COR-NMP protocol) were used in 16 declined human livers revealed that 11 of the originally declined organs met the viability criteria at the end of combined perfusion and were successfully transplanted. Transplanted livers showed a 100% graft survival after 6 months [47].

Thus, a combined perfusion technique consisting of HOPE followed by NMP not only attenuates IRI in livers in the course of transplantation, but also offers the possibility to assess declined livers for transplantation.

## 10. Additives and Targeted Treatment to Diminish IRI During MP

MP offers the unique possibility to administer specific additives, compounds, substances or drugs with an advantageous effect on the perfused organ. Treatment can be added directly to the perfusion solution in an adequate concentration. While passing through the vasculature, substances may exert a direct and exclusive effect on the organ perfused ex situ.

With regard to preventing IRI and restoring mitochondrial function during liver MP, the following candidates and concepts may be worth taking into consideration: Preventing succinate driven ROS accumulation may open the path to new therapies. A study in a heart IRI mouse model revealed a protective effect of inhibiting SDH against IRI [14]. Because opening the mPTP is a major regulator of cell death programs, the mPTP also represents a possible target for therapy. For example, the addition of cyclosporine, which inhibits formation of mPTP, could reduce IRI in a study with 58 patients suffering from acute myocardial infarction [70]. Moreover, there is evidence that the inhibition of caspases or the overexpression of anti-apoptotic proteins have beneficial effects to minimize IRI [71]. The process of necroptosis can be inhibited by the RIPK3 inhibitor necrostatin-1 (Nec-1), and in preclinical studies, a protective effect was demonstrated for Nec-1 therapy [24].

A determinant, which positively influences mitochondrial function during MP, is the supplement of an adequate oxygen carrier [72,73]. Matton et al. [72] performed a study using NMP in 12 discarded human livers and investigated the effect of a HBOC administered into the perfusate. The authors found similar ATP tissue levels upon perfusion with HBOC, compared to non-injured livers. Laing et al. [74] subjected discarded human livers to NMP, ensuring oxygen supply either by packed red blood cells or HBOC, and they report no positive effect on ATP tissue levels for HBOC. Moreover, they found no significant differences in ROS levels between the groups. In contrast, elevated ROS production has been described by others as one of the drawbacks of HBOC media, alongside increased vasoconstriction [69,74,75].

## 11. Conclusions

As IRI significantly determines graft function after transplantation, a profound understanding of the molecular mechanisms leading to tissue and cell injury are inevitable. An excessive mitochondrial response during ischemia fuels mROS production and decreases ATP levels, which have been identified as the major cause of reperfusion injury, leading to oxidative stress and tissue inflammation. Due to the high metabolic activity of hepatocytes, a liver graft is especially vulnerable to IRI. The technique of MP prior to transplantation may help to mitigate IRI in livers and serve as a platform for ex vivo organ therapy. The combination of HOPE followed by NMP holds great potential to effectively restore mitochondrial function and avoid redox stress.

## Figures and Tables

**Table 1:** Diverse preservation strategies of machine perfusion, depending on temperature and oxygen supply.

Perfusion Strategy	Temperature	Main Characteristics	Preservation Time (max)
HMP HOPE D-HOPE	4 °C	Reduced metabolism, ATP resynthesis, oxygen supplementation (HOPE, D-HOPE), perfusion via portal vein and hepatic artery (D-HOPE).	up to 24 h *
SNMP	20–25 °C	Sufficient metabolism for organ quality assessment, may combine advantages of HMP and NMP.	up to 24 h *
NMP	37 °C	Mimics in vivo conditions, organ quality assessment.	up to 7 d *
COR		Gradual rewarming → combination of the beneficial effects of different MP strategies.	

HMP: hypothermic machine perfusion, HOPE: hypothermic oxygenated machine perfusion, D-HOPE: dual hypothermic oxygenated perfusion, SNMP: subnormothermic machine perfusion, NMP: normothermic machine perfusion, COR: controlled oxygenated rewarming, ATP: adenosine triphosphate, * data obtained in studies with discarded human livers without subsequent transplantation.

## Abbreviations

ADP	Adenosine diphosphate
AIF	Apoptosis-inducing factor
ALT	Alanine aminotransferase
AST	Aspartate aminotransferase
ATP	Adenosine triphosphate
COR	Controlled oxygenated rewarming
CoQ	Coenzyme Q
DAMP	Damage associated molecular pattern
D-HOPE	Dual hypothermic oxygenated perfusion
ECD	Extended criteria donors
ET	Endothelin
ETS	Electron transport system
HBOC	Haemoglobin-based oxygen carrier
HDmiRNA	Hepatocyte-derived miRNA
HMGB-1	High mobility group-box 1 protein
HMP	Hypothermic machine perfusion
HOPE	Hypothermic oxygenated machine perfusion
HRR	High-resolution respirometry
HtrA2	High-temperature requirement protein A2
ICAM 1	Intracellular adhesion molecule 1
IL	Interkeukin
IMS	Intermembrane space
IRI	Ischemia/reperfusion injury
JAK/STAT	Janus kinase/signal transducers
KC	Kupffer cell
LSEC	Liver sinusoidal endothelial cell
LT	Liver transplantation
MAPK	Mitogen-activated protein kinase
miRNA	MicroRNA
MP	Machine perfusion
mPTP	Mitochondrial permeability transition pore
NAD/NADH	Nicotinamide adenine dinucleotide
NF-κB	Nuclear factor kappa B
NMP	Normothermic machine perfusion
NO	Nitric oxide
NOX	NADPH oxidases
RET	Reverse electron transport
RIPK1/RIPK3	Receptor-interacting protein kinases 1 and 3
ROS	Reactive oxygen species
mROS	Mitochondrial reactive oxygen species
SCS	Static cold storage
SDH	Succinate dehydrogenase
SNMP	Subnormothermic machine perfusion
TLR	Toll-like receptors
TNFR	Tumor necrosis factor receptor
TNFα	Tumor necrosis factor α
VCAM 1	Vascular adhesion molecule 1
XDH	Xanthine dehydrogenase
XO	Xanthine oxidase
XOR	Xanthine oxidoreductase

## References

[1] Adam R., Karam V., Delvart V., O’Grady J., Mirza D., Klempnauer J., Castaing D., Neuhaus P., Jamieson N., Salizzoni M. (2012). Evolution of indications and results of liver transplantation in Europe. A report from the European Liver Transplant Registry (ELTR). J. Hepatol..

[2] Schlegel A., Muller X., Dutkowski P. (2019). Machine perfusion strategies in liver transplantation. Hepatobiliary Surg. Nutr..

[3] Dar W.A., Sullivan E., Bynon J.S., Eltzschig H., Ju C. (2019). Ischaemia reperfusion injury in liver transplantation: Cellular and molecular mechanisms. Liver Int..

[4] Kalogeris T., Baines C.P., Krenz M., Korthuis R.J. (2012). Cell biology of ischemia/reperfusion injury. Int. Rev. Cell Mol. Biol..

[5] Kahn J., Schemmer P. (2018). Control of Ischemia-Reperfusion Injury in Liver Transplantation: Potentials for Increasing the Donor Pool. Visc. Med..

[6] Malhi H., Gores G.J. (2008). Cellular and molecular mechanisms of liver injury. Gastroenterology.

[7] Chouchani E.T., Pell V.R., James A.M., Work L.M., Saeb-Parsy K., Frezza C., Krieg T., Murphy M.P. (2016). A Unifying Mechanism for Mitochondrial Superoxide Production during Ischemia-Reperfusion Injury. Cell Metab..

[8] Zorov D.B., Juhaszova M., Sollott S.J. (2014). Mitochondrial reactive oxygen species (ROS) and ROS-induced ROS release. Physiol. Rev..

[9] Murphy E., Steenbergen C. (2008). Mechanisms underlying acute protection from cardiac ischemia-reperfusion injury. Physiol. Rev..

[10] Li N., Ragheb K., Lawler G., Sturgis J., Rajwa B., Melendez J.A., Robinson J.P. (2003). Mitochondrial complex I inhibitor rotenone induces apoptosis through enhancing mitochondrial reactive oxygen species production. J. Biol. Chem..

[11] Aldakkak M., Stowe D.F., Chen Q., Lesnefsky E.J., Camara A.K. (2008). Inhibited mitochondrial respiration by amobarbital during cardiac ischaemia improves redox state and reduces matrix Ca2+ overload and ROS release. Cardiovasc. Res..

[12] Eltzschig H.K., Eckle T. (2011). Ischemia and reperfusion—From mechanism to translation. Nat. Med..

[13] Bellini M.I., Yiu J., Nozdrin M., Papalois V. (2019). The Effect of Preservation Temperature on Liver, Kidney, and Pancreas Tissue ATP in Animal and Preclinical Human Models. J. Clin. Med..

[14] Chouchani E.T., Pell V.R., Gaude E., Aksentijevic D., Sundier S.Y., Robb E.L., Logan A., Nadtochiy S.M., Ord E.N.J., Smith A.C. (2014). Ischaemic accumulation of succinate controls reperfusion injury through mitochondrial ROS. Nature.

[15] Korge P., John S.A., Calmettes G., Weiss J.N. (2017). Reactive oxygen species production induced by pore opening in cardiac mitochondria: The role of complex II. J. Biol. Chem..

[16] Drose S., Bleier L., Brandt U. (2011). A common mechanism links differently acting complex II inhibitors to cardioprotection: Modulation of mitochondrial reactive oxygen species production. Mol. Pharm..

[17] Abramov A.Y., Scorziello A., Duchen M.R. (2007). Three distinct mechanisms generate oxygen free radicals in neurons and contribute to cell death during anoxia and reoxygenation. J. Neurosci..

[18] Eun H.S., Cho S.Y., Joo J.S., Kang S.H., Moon H.S., Lee E.S., Kim S.H., Lee B.S. (2017). Gene expression of NOX family members and their clinical significance in hepatocellular carcinoma. Sci. Rep..

[19] Sena L.A., Chandel N.S. (2012). Physiological Roles of Mitochondrial Reactive Oxygen Species. Mol. Cell.

[20] Kozlov A.V., Lancaster J.R., Meszaros A.T., Weidinger A. (2017). Mitochondria-meditated pathways of organ failure upon inflammation. Redox Biol..

[21] Ott M., Robertson J.D., Gogvadze V., Zhivotovsky B., Orrenius S. (2002). Cytochrome c release from mitochondria proceeds by a two-step process. Proc. Natl. Acad. Sci. USA.

[22] Reiners J.J., Caruso J.A., Mathieu P., Chelladurai B., Yin X.M., Kessel D. (2002). Release of cytochrome c and activation of pro-caspase-9 following lysosomal photodamage involves Bid cleavage. Cell Death Differ..

[23] Kilbride S.M., Prehn J.H. (2013). Central roles of apoptotic proteins in mitochondrial function. Oncogene.

[24] Linkermann A., Hackl M.J., Kunzendorf U., Walczak H., Krautwald S., Jevnikar A.M. (2013). Necroptosis in immunity and ischemia-reperfusion injury. Am. J. Transplant..

[25] Shi S., Verstegen M.M.A., Mezzanotte L., Jonge J., Löwik C.W.G.M., Laan L.J.W. (2019). Necroptotic Cell Death in Liver Transplantation and Underlying Diseases: Mechanisms and Clinical Perspective. Liver Transplant..

[26] Bhogal R.H., Weston C.J., Velduis S., Leuvenink H.G.D., Reynolds G.M., Davies S., Nyguet-Thin L., Alfaifi M., Shepard E.L., Boteon Y. (2018). The Reactive Oxygen Species-Mitophagy Signaling Pathway Regulates Liver Endothelial Cell Survival During Ischemia/Reperfusion Injury. Liver Transplant..

[27] Zhai Y., Petrowsky H., Hong J.C., Busuttil R.W., Kupiec-Weglinski J.W. (2013). Ischaemia-reperfusion injury in liver transplantation--from bench to bedside. Nat. Rev. Gastroenterol. Hepatol..

[28] Peralta C., Jimenez-Castro M.B., Gracia-Sancho J. (2013). Hepatic ischemia and reperfusion injury: Effects on the liver sinusoidal milieu. J. Hepatol..

[29] Roberts R.A., Ganey P.E., Ju C., Kamendulis L.M., Rusyn I., Klaunig J.E. (2007). Role of the Kupffer cell in mediating hepatic toxicity and carcinogenesis. Toxicol. Sci..

[30] Jing Y., Shishkov Andrei P.B.C. (2008). Inhibition of tumor necrosis factor alpha secretion in rat Kupffer cells by siRNA: In vivo efficacy of siRNA-liposomes. Biochim. Biophys. Acta.

[31] Lu T.F., Yang T.H., Zhong C.P., Shen C., Lin W.W., Gu G.X., Xia Q., Xu N. (2018). Dual Effect of Hepatic Macrophages on Liver Ischemia and Reperfusion Injury during Liver Transplantation. Immune Netw..

[32] Su L., Li N., Tang H., Lou Z., Chong X., Zhang C., Su J., Dong X. (2018). Kupffer cell-derived TNF-alpha promotes hepatocytes to produce CXCL1 and mobilize neutrophils in response to necrotic cells. Cell Death Dis..

[33] Dutkowski P., Guarrera J.V., de Jonge J., Martins P.N., Porte R.J., Clavien P.A. (2019). Evolving Trends in Machine Perfusion for Liver Transplantation. Gastroenterology.

[34] Weissenbacher A., Vrakas G., Nasralla D., Ceresa C.D.L. (2019). The future of organ perfusion and re-conditioning. Transplant. Int..

[35] Ceresa C.D.L., Nasralla D., Coussios C.C., Friend P.J. (2018). The case for normothermic machine perfusion in liver transplantation. Liver Transplant..

[36] Starzl T.E., Groth C.G., Brettschneider L., Moon J.B., Fulginiti V.A., Cotton E.K., Porter K.A. (1968). Extended survival in 3 cases of orthotopic homotransplantation of the human liver. Surgery.

[37] de Meijer V.E., Fujiyoshi M., Porte R.J. (2019). Ex situ machine perfusion strategies in liver transplantation. J. Hepatol..

[38] Schlegel A., Muller X., Dutkowski P. (2017). Hypothermic liver perfusion. Curr. Opin. Organ. Transplant..

[39] Schlegel A., Kron P., Dutkowski P. (2015). Hypothermic Oxygenated Liver Perfusion: Basic Mechanisms and Clinical Application. Curr. Transplant. Rep..

[40] Schlegel A., Kron P., Graf R., Clavien P.A., Dutkowski P. (2014). Hypothermic Oxygenated Perfusion (HOPE) downregulates the immune response in a rat model of liver transplantation. Ann. Surg..

[41] van Rijn R., Karimian N., Matton A.P.M., Burlage L.C., Westerkamp A.C., van den Berg A.P., de Kleine R.H.J., de Boer M.T., Lisman T., Porte R.J. (2017). Dual hypothermic oxygenated machine perfusion in liver transplants donated after circulatory death. Br. J. Surg..

[42] Bruggenwirth I.M.A., van Leeuwen O.B., de Vries Y., Bodewes S.B., Adelmeijer J., Wiersema-Buist J., Lisman T., Martins P.N., de Meijer V.E., Porte R.J. (2020). Extended hypothermic oxygenated machine perfusion enables ex situ preservation of porcine livers for up to 24 hours. JHep Rep..

[43] Karimian N., Raigani S., Huang V., Nagpal S., Hafiz E.O.A., Beijert I., Mahboub P., Porte R.J., Uygun K., Yarmush M. (2019). Subnormothermic Machine Perfusion of Steatotic Livers Results in Increased Energy Charge at the Cost of Anti-Oxidant Capacity Compared to Normothermic Perfusion. Metabolites.

[44] de Vries R.J., Tessier S.N., Banik P.D., Ozer S., Crorin S.E., Nagpal S., Yeh H., Uygun K. (2018). Extending the Human Liver Preservation Time for Transplantation by Supercooling. Transplantation.

[45] Eshmuminov D., Becker D., Bautista Borrego L., Hefti M., Schuler M.J., Hagedorn C., Muller X., Mueller M., Onder C., Graf R. (2020). An integrated perfusion machine preserves injured human livers for 1 week. Nat. Biotechnol.

[46] Minor T., Efferz P., Fox M., Wohlschlaeger J., Lüer B. (2013). Controlled oxygenated rewarming of cold stored liver grafts by thermally graduated machine perfusion prior to reperfusion. Am. J. Transplant..

[47] van Leeuwen O.B., de Vries Y., Fujiyoshi M., Nijsten M.W.N., Ubbink R., Pelgrim G.J., Werner M.J.M., Reyntjens K.M.E.M., van den Berg A.P., de Boer M.T. (2019). Transplantation of High-risk Donor Livers After Ex Situ Resuscitation and Assessment Using Combined Hypo- and Normothermic Machine Perfusion. Ann. Surg..

[48] Zhang Z.B., Gao W., Liu L., Shi Y., Ma N., Huai M.S., Shen Z.Y. (2019). Normothermic Machine Perfusion Protects Against Liver Ischemia-Reperfusion Injury During Reduced-Size Liver Transplantation in Pigs. Ann. Transplant..

[49] Schlegel A., Kron P., Graf R., Dutkowski P., Clavien P.A. (2014). Warm vs. cold perfusion techniques to rescue rodent liver grafts. J. Hepatol..

[50] Jassem W., Xystrakis E., Ghnewa Y.G., Yuksel M., Pop O., Martinez-Llordella M., Jabri Y., Huang X., Lozano J.J., Quaglia A. (2019). Normothermic Machine Perfusion (NMP) Inhibits Proinflammatory Responses in the Liver and Promotes Regeneration. Hepatology.

[51] Sutton M.E., op den Dries S., Karimian N., Weeder P.D., de Boer M.T., Wiersema-Buist J., Gouw A.S., Leuvenink H.G., Lisman T., Porte R.J. (2014). Criteria for viability assessment of discarded human donor livers during ex vivo normothermic machine perfusion. PLoS ONE.

[52] op den Dries S., Karimian N., Sutton M.E., Westerkamp A.C., Nijsten M.W., Gouw A.S., Wiersema-Buist J., Lisman T., Leuvenink H.G., Porte R.J. (2013). Ex vivo normothermic machine perfusion and viability testing of discarded human donor livers. Am. J. Transplant..

[53] Nasralla D., Coussios C.C., Mergental H., Akhtar M.Z., Butler A.J., Ceresa C.D.L., Chiocchia V., Dutton S.J., Garcia-Valdecasas J.C., Heaton N. (2018). A randomized trial of normothermic preservation in liver transplantation. Nature.

[54] Watson C.J.E., Kosmoliaptsis V., Randle L.V., Gimson A.E., Brais R., Klinck J.R., Hamed M., Tsyben A., Butler A.J. (2017). Normothermic Perfusion in the Assessment and Preservation of Declined Livers Before Transplantation. Transplantation.

[55] Matton A.P.M., de Vries Y., Burlage L.C., van Rijn R., Fujiyoshi M., de Meijer V.E., de Boer M.T., de Kleine R.H.J., Verkade H.J., Gouw A.S.H. (2019). Biliary Bicarbonate, pH, and Glucose Are Suitable Biomarkers of Biliary Viability During Ex Situ Normothermic Machine Perfusion of Human Donor Livers. Transplantation.

[56] de Vries R.J., Pendexter C.A., Cronin S.E.J., Marques B., Hafiz E.O.A., Muzikansky A., van Gulik T.M., Markmann J.F., Stott S.L., Yeh H. (2020). Cell release during perfusion reflects cold ischemic injury in rat livers. Sci. Rep..

[57] Karangwa S.A., Burlage L.C., Adelmeijer J., Karimian N., Westerkamp A.C., Matton A.P., van Rijn R., Wiersema-Buist J., Sutton M.E., Op den Dries S. (2017). Activation of Fibrinolysis, But Not Coagulation, During End-Ischemic Ex Situ Normothermic Machine Perfusion of Human Donor Livers. Transplantation.

[58] Matton A.P.M., Selten J.W., Roest H.P., de Jonge J., IJzermans J.N.M., de Meijer V.E., Porte R.J., van der Laan L.J.W. (2020). Cell-free microRNAs as early predictors of graft viability during ex vivo normothermic machine perfusion of human donor livers. Clin. Transplant..

[59] Garcia-Roche M., Casal A., Carriquiry M., Radi R., Quijano C., Cassina A. (2018). Respiratory analysis of coupled mitochondria in cryopreserved liver biopsies. Redox Biol..

[60] Kuznetsov A.V., Strobl D., Ruttmann E., Konigsrainer A., Margreiter R., Gnaiger E. (2002). Evaluation of mitochondrial respiratory function in small biopsies of liver. Anal. Biochem..

[61] Chu M.J., Phillips A.R., Hosking A.W., MacDonald J.R., Bartlett A.S., Hickey A.J. (2013). Hepatic mitochondrial function analysis using needle liver biopsy samples. PLoS ONE.

[62] Kappler L., Hoene M., Hu C., von Toerne C., Li J., Bleher D., Hoffmann C., Bohm A., Kollipara L., Zischka H. (2019). Linking bioenergetic function of mitochondria to tissue-specific molecular fingerprints. Am. J. Physiol. Endocrinol. Metab..

[63] Ost M., Doerrier C., Gama-Perez P., Moreno-Gomez S. (2018). Analysis of mitochondrial respiratory function in tissue biopsies and blood cells. Curr. Opin. Clin. Nutr. Metab. Care.

[64] Chu M.J., Premkumar R., Hickey A.J., Jiang Y., Delahunt B., Phillips A.R., Bartlett A.S. (2016). Steatotic livers are susceptible to normothermic ischemia-reperfusion injury from mitochondrial Complex-I dysfunction. World J. Gastroenterol..

[65] Doerrier C., Garcia-Souza L.F., Krumschnabel G., Wohlfarter Y., Meszaros A.T., Gnaiger E. (2018). High-Resolution FluoRespirometry and OXPHOS Protocols for Human Cells, Permeabilized Fibers from Small Biopsies of Muscle, and Isolated Mitochondria. Methods Mol. Biol..

[66] Pesta D., Gnaiger E. (2012). High-resolution respirometry: OXPHOS protocols for human cells and permeabilized fibers from small biopsies of human muscle. Methods Mol. Biol..

[67] Boteon Y.L., Laing R.W., Schlegel A., Wallace L., Smith A., Attard J., Bhogal R.H., Neil D.A.H., Hubscher S., Perera M. (2018). Combined Hypothermic and Normothermic Machine Perfusion Improves Functional Recovery of Extended Criteria Donor Livers. Liver Transplant..

[68] Martins R.M., Teodoro J.S., Furtado E., Oliveira R.C., Tralhao J.G., Rolo A.P., Palmeira C.M. (2019). Mild hypothermia during the reperfusion phase protects mitochondrial bioenergetics against ischemia-reperfusion injury in an animal model of ex-vivo liver transplantation-an experimental study. Int. J. Med. Sci..

[69] Boteon Y.L., Laing R.W., Schlegel A., Wallace L., Smith A., Attard J., Bhogal R.H., Reynolds G., Perera M., Muiesan P. (2019). The impact on the bioenergetic status and oxidative-mediated tissue injury of a combined protocol of hypothermic and normothermic machine perfusion using an acellular haemoglobin-based oxygen carrier: The cold-to-warm machine perfusion of the liver. PLoS ONE.

[70] Piot C., Croisille P., Staat P., Thibault H., Rioufol G., Mewton N., Elbelghiti R., Tri Chung T., Bonnefoy E., Angoulvant D. (2008). Effect of Cyclosporine on Reperfusion Injury in Acute Myocardial Infarction. N. Engl. J. Med..

[71] Murphy M.P. (2016). Understanding and preventing mitochondrial oxidative damage. Biochem. Soc. Trans..

[72] Matton A.P.M., Burlage L.C., van Rijn R., de Vries Y., Karangwa S.A., Nijsten M.W., Gouw A.S.H., Wiersema-Buist J., Adelmeijer J., Westerkamp A.C. (2018). Normothermic machine perfusion of donor livers without the need for human blood products. Liver Transplant..

[73] de Vries Y., Matton A.P.M., Nijsten M.W.N., Werner M.J.M., van den Berg A.P., de Boer M.T., Buis C.I., Fujiyoshi M., de Kleine R.H.J., van Leeuwen O.B. (2019). Pretransplant sequential hypo- and normothermic machine perfusion of suboptimal livers donated after circulatory death using a hemoglobin-based oxygen carrier perfusion solution. Am. J. Transplant..

[74] Laing R.W., Bhogal R.H., Wallace L., Boteon Y., Neil D.A.H., Smith A., Stephenson B.T.F., Schlegel A., Hubscher S.G., Mirza D.F. (2017). The Use of an Acellular Oxygen Carrier in a Human Liver Model of Normothermic Machine Perfusion. Transplantation.

[75] Eshmuminov D., Leoni F., Schneider M.A., Becker D., Muller X., Onder C., Hefti M., Schuler M.J., Dutkowski P., Graf R. (2018). Perfusion settings and additives in liver normothermic machine perfusion with red blood cells as oxygen carrier. A systematic review of human and porcine perfusion protocols. Transplant. Int..

